# Availability of Family Caregiver Programs in US Cancer Centers

**DOI:** 10.1001/jamanetworkopen.2023.37250

**Published:** 2023-10-11

**Authors:** J. Nicholas Odom, Allison Applebaum, Marie A. Bakitas, Tara Bryant, Erin Currie, Kayleigh Curry, Heidi Donovan, Maria E. Fernandez, Betty Ferrell, Andres Azuero, Tamryn F. Gray, Bailey A. Hendricks, Diane Meier, Chandylen Nightingale, Susan Reinhard, Timothy S. Sannes, Katherine Sterba, Heather M. Young

**Affiliations:** 1 School of Nursing, The University of Alabama at Birmingham, Birmingham; 2 Division of Geriatrics, Gerontology, and Palliative Care, Department of Medicine, The University of Alabama at Birmingham, Birmingham; 3 Center for Palliative and Supportive Care, The University of Alabama at Birmingham, Birmingham; 4 Department of Psychiatry and Behavioral Sciences, Memorial Sloan Kettering Cancer Center, New York, New York; 5 Viva Health Inc, Birmingham, Alabama; 6 School of Nursing, University of Pittsburgh, Pittsburgh, Pennsylvania; 7 School of Medicine, University of Pittsburgh, Pittsburgh, Pennsylvania; 8 National Rehabilitation Research and Training Center on Family Support, University of Pittsburgh, Pittsburgh, Pennsylvania; 9 Department of Health Promotion and Behavioral Sciences, University of Texas Health Science Center at Houston School of Public Health, Houston; 10 City of Hope, Duarte, California; 11 Department of Psychosocial Oncology and Palliative Care, Dana-Farber Cancer Institute, Harvard Medical School, Boston, Massachusetts; 12 College of Nursing, University of Nebraska Medical Center, Omaha; 13 Mount Sinai Medical Center, New York, New York; 14 Department of Social Sciences and Health Policy, School of Medicine, Wake Forest University, Winston-Salem, North Carolina; 15 AARP Public Policy Institute, Washington, DC; 16 UMass Memorial Cancer Center, Worcester, Massachusetts; 17 Department of Public Health Sciences, Medical University of South Carolina, Charleston; 18 Betty Irene Moore School of Nursing, University of California, Davis

## Abstract

**Importance:**

Family caregivers provide the majority of health care to the 18 million patients with cancer in the US. Yet despite providing complex medical and nursing care, a large proportion of caregivers report no formal support or training. In recognition of this gap, many interventions to support cancer caregivers have been developed and tested over the past 2 decades. However, there are few system-level data on whether US cancer centers have adopted and implemented these interventions.

**Objective:**

To describe and characterize the availability of family caregiver support programs in US cancer centers.

**Design, Setting, and Participants:**

This cross-sectional national survey study was conducted between September 1, 2021, and April 30, 2023. Participants comprised clinical and administrative staff of Commission on Cancer–accredited US cancer centers. Data analysis was performed in May and June 2023.

**Main Outcomes and Measures:**

Survey questions about the availability of 11 types of family caregiver programs (eg, peer mentoring, education classes, and psychosocial programs) were developed after literature review, assessment of similar program evaluation surveys, and discussions among a 13-member national expert advisory committee. *Family caregiver programs* were defined as structured, planned, and coordinated groups of activities and procedures aimed at specifically supporting family caregivers as part of usual care. Survey responses were tabulated using standard descriptive statistics, including means, proportions, and frequencies.

**Results:**

Of the surveys sent to potential respondents at 971 adult cancer centers, 238 were completed (response rate, 24.5%). After nonresponse weight adjustment, most cancer centers (75.4%) had at least 1 family caregiver program; 24.6% had none. The most common program type was information and referral services (53.6%). Cancer centers with no programs were more likely to have smaller annual outpatient volumes (χ^2^ = 11.10; *P* = .011). Few centers had caregiver programs on training in medical and/or nursing tasks (21.7%), caregiver self-care (20.2%), caregiver-specific distress screening (19.3%), peer mentoring (18.9%), and children caregiving for parents (8.3%). Very few programs were developed from published evidence in a journal (8.1%). The top reason why cancer centers selected their programs was community members requesting the program (26.3%); only 12.3% of centers selected their programs based on scientific evidence. Most programs were funded by the cancer center or hospital (58.6%) or by philanthropy (42.4%).

**Conclusions and Relevance:**

In this survey study, most cancer centers had family caregiver programs; however, a quarter had none. Furthermore, the scope of programming was limited and rarely evidence based, with few centers offering caregiving education and training. These findings suggest that implementation strategies are critically needed to foster uptake of evidence-based caregiver interventions.

## Key Points

**Question:**

What are the availability and characteristics of family caregiver support programs in US cancer centers?

**Findings:**

In this survey study of 238 Commission on Cancer–accredited US cancer centers, most had family caregiver programs; however, a quarter had none. The scope of programming was limited and rarely evidence based.

**Meaning:**

These findings suggest that implementation strategies are critically needed to foster uptake of evidence-based cancer caregiver interventions.

## Teaser

This survey study examines the availability and characteristics of family caregiver support programs offered by Commission on Cancer–accredited cancer centers in the US.

## Introduction

Unpaid family and friend caregivers provide the majority of health care to the 18 million individuals with cancer in the US. Yet despite providing complex medical and nursing care, a large proportion of caregivers receive little to no formal support or training.^1,2^ Cancer caregivers can provide up to an average of 8 hours of daily assistance to care recipients,^3^ including managing and monitoring symptoms, coordinating care, communicating with the health care team, partnering in health care decision making, managing medications, and offering psychological and emotional support.^1,4,5^ In recognition of this wide variation in and complexity of caregiver tasks, legislative action such as the CARE (Caregiver Advise, Record, Enable) Act has been passed in most US states (42 as of this writing), requiring hospitals to provide education to family caregivers on medical and nursing tasks when transitioning from hospital to home.^6^ However, population-based estimates^7^ have reported that although 72% of cancer caregivers assist with medical or nursing tasks (eg, administering injections, tube feedings, colostomy care), 43% do so without any prior formal preparation.

Recognizing the lack of and critical need for caregiver support, numerous effective interventions to train cancer caregivers have been developed and tested over the past 2 decades. Systematic reviews and meta-analyses of cancer caregiver support interventions have cataloged approximately 75 interventions that have been developed and tested.^8,9^ Furthermore, these interventions have been found to positively affect both caregiver and patient outcomes.^10,11,12^ Yet despite development of effective caregiver interventions, there are few system-level data on whether they have been adopted and implemented in US cancer centers. A prior study by Nightingale et al^13^ examined caregiver engagement practices, including supportive care service availability, in 204 National Cancer Institute Community Oncology Research Program (NCORP) community oncology practice groups. The investigators found that 64% had some type of supportive care service available to caregivers.

To extend and broaden this system-level assessment of cancer caregiver support beyond community oncology groups, we conducted a national survey of Commission on Cancer (CoC)–accredited US cancer centers to characterize and determine the availability of family caregiver support programs. We aimed to examine (1) the proportion of cancer centers offering adult caregiver–specific programs and services, including types of programs; (2) why cancer centers selected their caregiver programs; (3) how these programs were developed; and (4) how these programs were funded. Evaluating these questions at a national level is timely because new caregiving policy initiatives, such as President Biden’s 2023 Executive Order on Increasing Access to High-Quality Care and Supporting Caregivers, are being unveiled.^14^ Such policy and system strategies that advance caregiver support ultimately need grounding in national-level data, which is a further impetus for this work.^2^

## Methods

### Survey Development

The University of Alabama at Birmingham (UAB) Institutional Review Board approved the protocol for this survey study, including an information sheet and a waiver of signed informed consent. The study followed the American Association for Public Opinion Research (AAPOR) reporting guideline.

The survey was based conceptually on the Donabedian Model of Health Care Systems and its 3-component quality care model (ie, structure, process, and outcomes).^15,16^ Items were generated after a comprehensive literature review, including adapting items from similar surveys,^13,17,18^ and refined through feedback and discussion among a 13-member national expert advisory panel comprising cancer family caregiving research and policy experts (6 nurses, 2 physicians, 2 clinical psychologists, and 3 health services researchers). A medical oncologist, a palliative care physician, and a clinical psychologist associated with the UAB Center for Palliative and Supportive Care provided additional feedback. The survey was subsequently field-tested for readability, face validity, and survey completion time with 2 psycho-oncology counseling clinicians. The final survey (eAppendix in Supplement 1) consisted of 19 items asking about cancer center and family caregiver program details, including types of programs offered, how they were developed and funded, and the support needed to develop caregiver programs. The Box presents the verbatim language used to define the term *family caregiver program* in the survey. This term was defined to focus on caregiver-focused programs (1) that were part of usual care (ie, not research) and (2) that reflected evidence-based programs and not simply the routine practices of clinicians (eg, social workers, navigators).

Box. **Definition of *Family Caregiver Program* Used in the Survey**

For the purposes of this survey, we define a family caregiver program as a structured, planned, coordinated group of activities and procedures aimed at specifically supporting family caregivers as part of usual care in your cancer center. A program can be aimed at helping caregivers in their role supporting patients and/or in taking care of themselves. A program can be one that is offered only for specific cancers (eg, a support group for caregivers of patients with brain tumors).

Importantly, we do not consider the following to be a family caregiver program:

- A program wholly funded by a research study or time-limited quality improvement project.
- Simply having social workers or navigators employed at your cancer center.
- A clinical referral pathway for distressed caregivers that provides services that bills their health insurance (ie, they become a “patient” with a medical record number in the health system).
- A program focused primarily on patients. A family caregiver program might include patient participation but needs to include the family caregiver as an essential participant in the program.

### Cancer Centers

US cancer centers were identified through the publicly available CoC online database.^19^ The American College of Surgeons founded the CoC in 1922 to establish standards and accredit cancer centers who meet 34 quality standards. The CoC-accredited cancer centers represent 30% of all US hospitals and approximately 70% of patients with cancer. After pediatric cancer centers were excluded for the purposes of this study, an initial list of 1311 cancer centers was obtained.

### Survey Process and Response Rate Calculation

The names and contact information for potential cancer center clinician and administrator respondents who would potentially have “a general knowledge of support services and programs for family caregivers” in their cancer center were obtained by several approaches. The first was a review of cancer center websites to identify listed personnel who had email or mailing address information. A list of potential contacts was also provided by IQVIA, a health care data analytics company that provides clinical research and development services to cancer centers in both the US and internationally. Contacts were also garnered from the professional contact networks of our 13-member national expert advisory panel. Using a modified Dillman survey approach,^20^ potential respondents were either mailed or emailed a series of correspondences with the survey, including reminders, that could be completed by mail or online through a survey link. Data collection occurred from September 1, 2021, and April 30, 2023. Individuals completing the survey were offered a $20 incentive.

Response rates were determined using AAPOR metrics.^21^ The formula for calculation was as follows: Response rate = (Complete Responses + Partial Responses)/(Complete Responses + Partial Responses + Nonresponse + Explicit Refusals + Implicit Refusals). For this study, a complete response was defined as 80% of questions answered or more; a partial response was defined as less than 80% of questions answered plus completion of the item pertaining to the presence of family caregiver support services. Cancer centers were not included in the analysis (n = 340) if we were unable to identify or reach a potential respondent who could have completed the survey.

### Statistical Analysis

SPSS, version 29.0 (IBM Corp), was used for all analyses. Responding and nonresponding cancer centers were compared by US region and rural-urban status (defined by US Census Rural-Urban Commuting Area Codes),^22^ as this information was discernable from cancer center zip codes. To adjust for observed small differences, nonresponse adjustment weights^23^ were estimated by stabilized inverse probability weights,^24^ which were computed with a logistic regression model for survey response (yes vs no) using US region, rural-urban status, and an interaction between these 2 as estimators. Among responders, the stabilized weights were estimated as *w_i_* = *P*(*responders*)/*e_i_*, where *w_i_* is the weight for center *i*, *P*(*responders*) is the overall proportion of responders, and *e_i_* is the probability of response for center *i* estimated by the logistic regression model. Among responders, the sum of weights was checked to match with the original frequency of responders, and the weighted proportions by US region and rural-urban status were checked to match with the overall proportions. The weights were used on all subsequent analyses.

**Figure 1.**
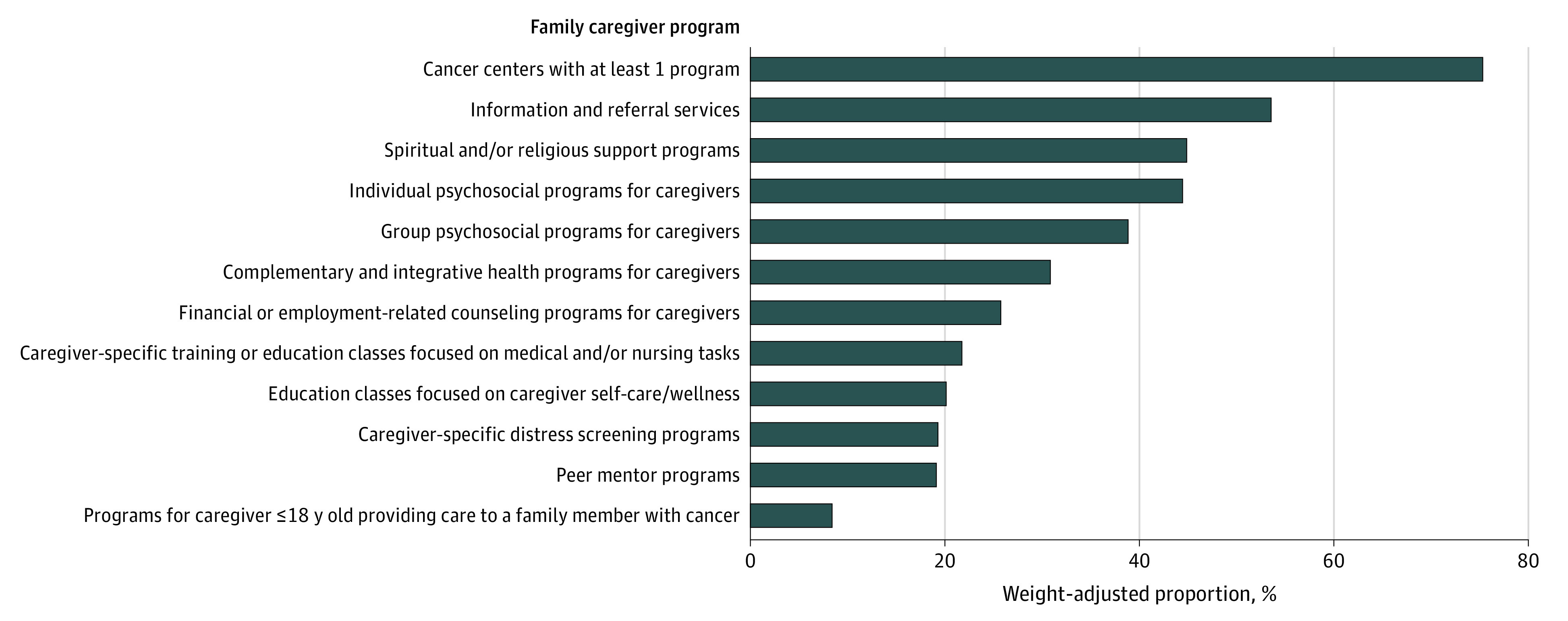
Weight-Adjusted Proportion of US Cancer Centers Offering Family Caregiver Programs (N = 238)

The primary outcome was having at least 1 of 11 different types of family caregiver programs, listed in Figure 1. Survey responses were tabulated using standard descriptive statistics, including means, proportions, and frequencies. We estimated the prevalence of caregiver programs nationally by extrapolating from the proportion of participating cancer centers that reported having programs. A χ^2^ test was used to examine the association between having at least 1 caregiver program and cancer center characteristics, including survey respondent–reported annual outpatient volume, number of oncologists, type of ownership, and US region.

Statistical significance was determined at *P* < .05 (2 tailed). Data analysis was performed in May and June 2023.

## Results

### Response Rates and Responder Characteristics

Of the 971 cancer centers with identified contacts, 238 completed the survey (response rate: 24.5%; survey study flow in eFigure 1 in Supplement 1). Characteristics of survey responders and cancer centers are presented in the Table. Participating cancer centers represented 43 US states and did not differ from nonparticipating centers by US region (χ^2^ = 4.42; *P* = .22). However, they did differ by rural-urban status, with a slight overrepresentation by rural cancer centers (χ^2^ = 5.66; *P* = .02; Cramer *V* = 0.08) (eTable in Supplement 1).

**Table.** Characteristics of Survey Responders and Cancer Centers

Characteristic	No. of cancer center respondents (%) (N = 238)
Respondent role	
Nurse	78 (32.8)
Social worker	58 (24.4)
Nurse practitioner	38 (16.0)
Physician	33 (13.9)
Health care administrator, coordinator, or director	17 (7.1)
Other (eg, behavioral health counselor, health educator, physician assistant, psychologist)	13 (2.5)
Missing	1 (0.4)
No. of y employed at cancer center	
≤1	11 (4.6)
2-5	82 (34.5)
6-10	64 (26.9)
11-15	31 (13.0)
16-20	16 (6.7)
≥21	32 (13.4)
Missing	2 (0.8)
No. of cancer center outpatients^a^	
<1000	44 (18.5)
1000-5000	80 (33.6)
5000-10 000	39 (16.4)
10 000-15 000	12 (5.0)
15 000-20 000	15 (6.3)
>20 000	18 (7.6)
Missing	30 (12.6)
Total No. of oncologists	
≤5	82 (34.5)
6-10	72 (30.3)
11-15	35 (14.7)
16-20	12 (5.0)
21-25	10 (4.2)
≥26	25 (10.5)
Missing	2 (0.8)
Cancer center ownership	
Independently owned (ie, single hospital or small regional network [≤3 hospitals] or an independent clinic/physician practice)	65 (27.3)
Hospital, clinic, or physician practice owned by a large regional or multistate health system that includes a health plan	96 (40.3)
Hospital, clinic, or physician practice owned by a large regional or multistate health system that does not include a health plan	32 (13.4)
Publicly owned (eg, state, county, or city)	15 (6.3)
Academic medical center	25 (10.5)
Other	3 (1.3)
Missing	2 (0.8)
Geography	
Rural	195 (81.9)
Urban	43 (18.1)
US region	
West	47 (19.7)
Midwest	70 (29.4)
Northeast	50 (21.0)
South	71 (29.8)

^a^ Number categories overlap in accordance with the survey.

### Availability of Family Caregiver Programs

Figure 1 provides an overview of weight-adjusted, respondent-reported family caregiver programs at US cancer centers, including by type. Of the 238 cancer centers, 75.4% had at least 1 family caregiver program; 24.6% had none. The 3 most common programs were information and referral services (53.6%), spiritual and/or religious support programs (44.9%), and individual psychosocial programs for caregivers (44.4%). The least commonly reported programs were caregiver-specific training or education classes focused on medical and/or nursing tasks (21.7%), education classes focused on caregiver self-care and/or wellness (20.2%), caregiver-specific distress screening programs (19.3%), peer mentor programs (18.9%), and programs for caregivers aged 18 years or younger providing care to a family member with cancer (8.3%).

In χ^2^ analyses, there was a statistically significant relationship between the number of annual cancer center outpatients and the presence of at least 1 family caregiver program (χ^2^ = 11.10; *P* = .011; Cramer *V* = 0.23). Cancer centers with smaller annual outpatient volumes were less likely to have a caregiver program. There were no other statistically significant differences for centers having at least 1 program and all other cancer center characteristics, including total number of oncologists, cancer center ownership, or US region.

### Reasons for Program Selection, Sources of Development, and Funding

**Figure 2.**
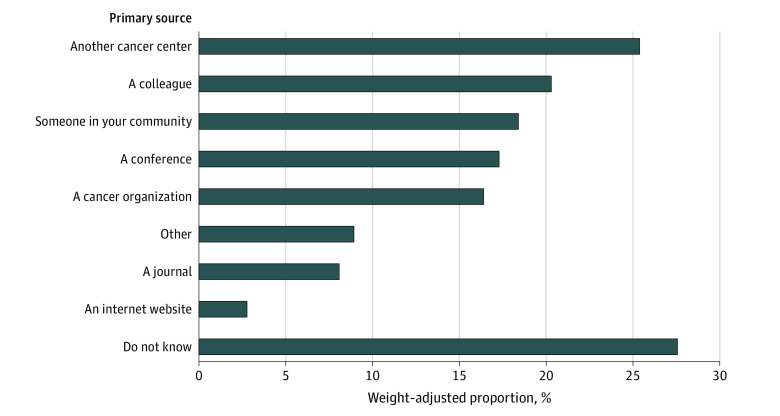
Weight-Adjusted Primary Sources Influencing Development of Family Caregiver Programs (N = 238) For this survey item, respondents were asked to “check all that apply.”

Figure 2 presents the weight-adjusted primary sources influencing development of cancer center family caregiver programs. Nearly a third of respondents did not know the sources influencing the development of their programs (27.6%). A quarter of cancer centers (25.4%) reported that their program or programs were influenced by another cancer center, followed by a colleague (20.3%) and someone in their community (18.4%). Only 8.1% reported that their program was influenced by published evidence in a journal.

**Figure 3.**
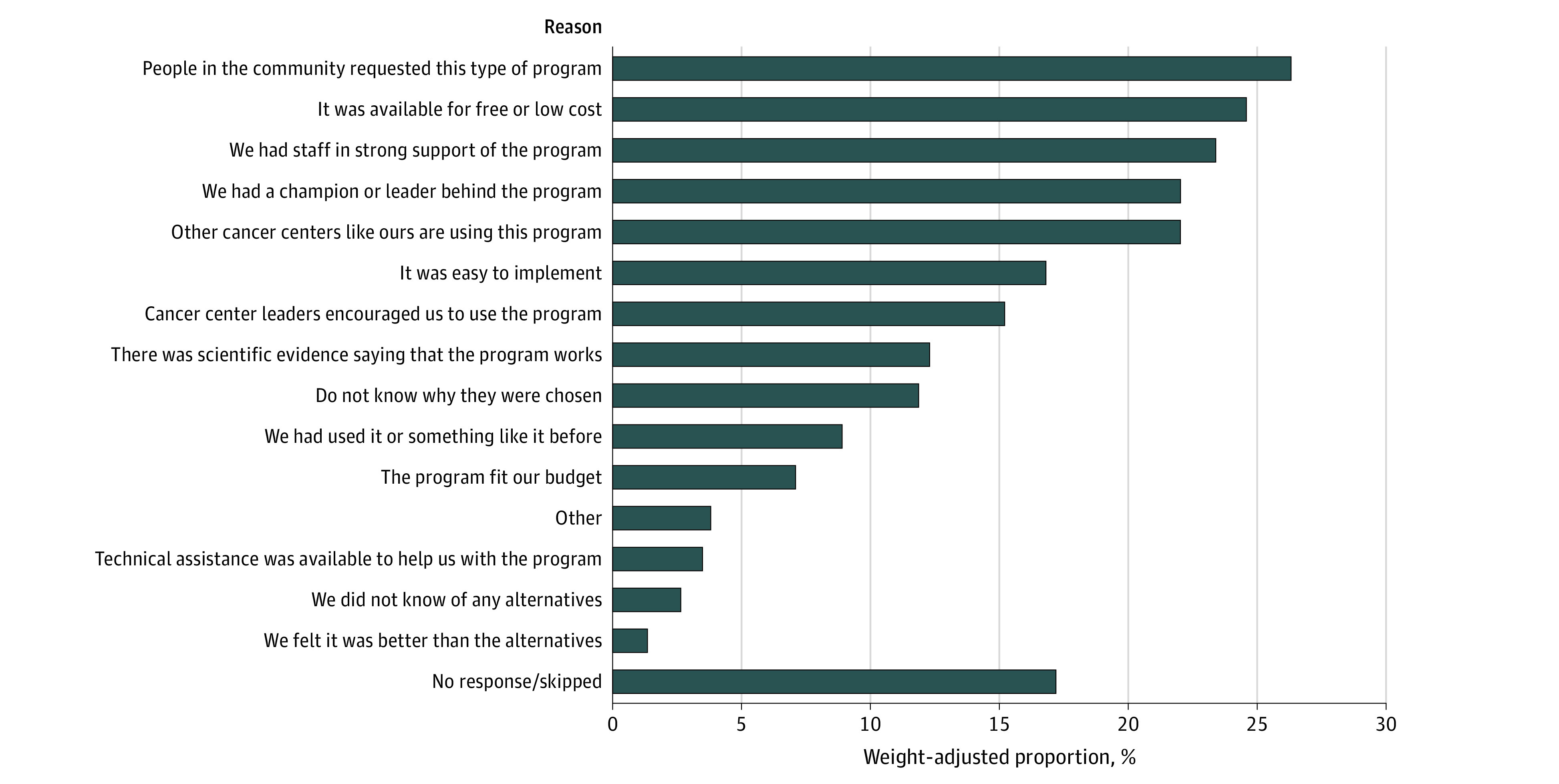
Weight-Adjusted Reasons Why Family Caregiver Programs Were Chosen (N = 238)

Figure 3 lists the weight-adjusted reasons why cancer centers chose family caregiver programs. The top reasons included community members requesting the program (26.3%), it being available for free or low cost (24.6%), having staff (23.4%) and/or a champion or leader (22.0%) in support of the program, and other similar cancer centers using the program (22.0%). Only 12.3% reported that their program was chosen because “there was scientific evidence saying the program works.”

eFigure 2 in Supplement 1 presents the weight-adjusted primary sources that financially supported cancer center family caregiver programs. Most respondents reported that their programs were supported by the cancer center or the hospital (58.6%). Almost half (42.4%) reported that a philanthropic or other individual or community donation supported their program or programs.

## Discussion

We conducted a national survey of CoC-accredited US cancer centers to report the current availability of programs to support family caregivers of patients with cancer. Despite the rapidly increasing number of evidence-based cancer caregiver interventions tested and published over the past 3 decades^8,9^ and the increasing spotlight on the critical need to support caregivers by the National Academies of Sciences, Engineering, and Medicine and policies like the CARE Act,^2,25^ a quarter of US cancer centers in our sample (24.6%) did not have any caregiver programming. Of the cancer centers in this study who had programs, the scope of programming was generally limited to information and referral services and proportionally few cancer centers offered caregiver education and training programs. Because very few programs were selected based on scientific evidence and developed from a journal in which evidence-based caregiving interventions were reported, new strategies are needed to improve the dissemination, adoption, and implementation of evidence-based cancer caregiving interventions into clinical practice.

Three-quarters of cancer centers in our survey sample (75.4%) had at least 1 family caregiver program. This is marginally higher but generally consistent with findings by Nightingale et al,^13^ who reported that 64% of NCORP community oncology practice groups had some type of “supportive care services” for families. Although most cancer centers included in our study had some type of formal support for at least some of their caregivers, only 8.1% reported that their program was influenced by evidence in a journal and only 12.3% chose their program because there was scientific evidence of its effectiveness. These findings suggest that uptake and implementation of existing evidence-based cancer caregiver interventions is extremely limited. Furthermore, it is unclear from our data how accessible the caregiver programs were, how many caregivers are served, and how equitably the services are delivered. Future research should seek to ascertain not only the existence of caregiving programs but also their uptake and penetrance.

Among the challenges cancer centers face in implementing family caregiver support, major barriers are the lack of payment structures and reimbursement mechanisms to support programs for families, the lack of quality metrics and accreditation standards focused on caregivers, and the lack of an available workforce to deliver support.^2,26^ Indeed, we observed that cancer centers that were smaller in size (as indicated by smaller annual outpatient volumes) were less likely to have caregiver programs, which we suspect is in part due to having fewer financial resources and personnel. It should also be noted that this survey study was administered during the height of the COVID-19 pandemic, which may have limited the personnel and resources available to support caregiver programming.

Only 1 in 5 cancer centers in this study had programs focused on caregiver self-care and caregiver training on medical and/or nursing tasks. These findings are troubling for 2 reasons. First, cancer caregivers have been shown to have poor self-care, often sacrificing their own health and experiencing distress to support their care recipients.^27^ Second, administration of some cancer treatments, including hematopoietic cell transplantation, chimeric antigen receptor T-cell therapy, and infusion chemotherapy, is increasingly shifting to the home, thereby increasing reliance on family caregivers.^28,29^ Furthermore, although 72% of cancer caregivers perform complex medical and/or nursing tasks, less than half report any preparation.^1^ Hence, we believe it is imperative that programmatic development in US cancer centers should focus on these 2 key areas to best meet the needs of both caregivers and patients.

Our findings have 4 major implications. First, national strategies are needed that focus on assisting cancer centers with implementing and sustaining cancer caregiver support that is evidence based. Such a strategy might include tools, resources, and technical assistance to help individual cancer centers with implementation of caregiving programs and in partnership with their local patient and family communities to ensure cultural fit and uptake.^30^ Second, consistent with CARE Act mandates, systematic screening and identification of caregivers is needed to improve surveillance of this population at a public health level to both better understand this “hidden” oncology workforce and to match programs and services to those whose needs are greatest.^31^ Third, policy and business model solutions are needed to incentivize cancer centers to provide effective, fiscally sustainable caregiver programming. A quarter of caregiver programs (24.6%) in this study were offered because they were free or low cost, suggesting that being able to pay for programming was a pressing consideration. Furthermore, 42.4% of programs were supported by philanthropy, potentially indicating an overreliance on outside financial support to be able to provide caregiver support. Hence, reliable business models could ensure fiscal support for vital programming. Finally, researchers who develop and test cancer caregiver interventions need to prioritize affordability, efficiency, and scalability at the same level of importance as effectiveness because many cancer caregiver interventions have been observed to be difficult to implement.^32^

### Limitations

There are several limitations to this study. First, our 24.5% response rate may raise concern about generalizability. However, our nonresponder analyses suggested no differences between responding and nonresponding cancer centers by US region and only small differences by rural-urban status. Furthermore, all results had a nonresponse weight adjustment. The lower-than-anticipated response rate may have been due to survey administration during the height of the COVID-19 pandemic, when clinicians and staff were extraordinarily burdened with patient care. Second, we attempted to identify individuals who self-endorsed having a general knowledge of caregiving services in the cancer center. However, it is possible that some respondents may not have been completely knowledgeable about or up-to-date with programs in their cancer center. To address this, the survey instructions encouraged individuals to ask for help from their work colleagues to answer the survey or to refer the study team to another individual in the cancer center who might be better suited to complete it. Third, cancer centers with caregiver programs and champions to support them may have been more likely to respond to the survey. This may have resulted in an overestimation of the proportion of centers with programs reported here. Fourth, there is no gold-standard definition of a family caregiver program. Although we provided participants with a specific definition of this term (Box), some endorsed caregiver programs may have represented minimal offerings (eg, information and referral services) not meeting our comprehensive definition, thereby inflating the actual number of cancer centers with programs. In future work, investigators should be mindful to be very specific in how they are defining caregiver programs and other types of support.

## Conclusions

In this survey study of 238 CoC-accredited US cancer centers, we observed that most centers had some type of family caregiver program; however, a quarter had none. Furthermore, the scope of programming was primarily limited to information and referral services for caregivers and was rarely evidence based, with few centers offering education and training. Given the growing recognition of family caregivers as central to the oncology workforce, it is imperative to develop national strategies and policy to accelerate the implementation of evidence-based caregiver support into practice.

## Notes

Supplement 1. **eAppendix.** Final Survey **eFigure 1.** Study Flow Diagram **eFigure 2.** Weight-Adjusted Primary Sources of Funding for Family Caregiver Programs (N = 238) **eTable.** Nonresponding vs Responding Cancer Center Characteristics

Supplement 2.
Data Sharing Statement
