# Supplementary material for: Availability of Family Caregiver Programs in US Cancer Centers
Source: JAMA Netw Open. 2023 Oct 11;6(10):e2337250. doi: 10.1001/jamanetworkopen.2023.37250 (PMC10568368; doi:10.1001/jamanetworkopen.2023.37250)
Supplement: Supplement 1. — eAppendix. Final Survey eFigure 1. Study Flow Diagram eFigure 2. Weight-Adjusted Primary Sources of Funding for Family Caregiver Programs (N = 238) eTable. Nonresponding vs Responding Cancer Center Characteristics [file jamanetwopen-e2337250-s001.pdf]

Supplementary Online Content

Odom JN, Applebaum A, Bakitas MA, et al. Availability of family caregiver programs in US cancer centers. *JAMA Netw Open*. 2023;6(10):e2337250. doi:10.1001/jamanetworkopen.2023.37250

eAppendix. Final Survey

eFigure 1. Study Flow Diagram

eFigure 2. Weight-Adjusted Primary Sources of Funding for Family Caregiver Programs (N = 238)

eTable. Nonresponding vs Responding Cancer Center Characteristics

This supplementary material has been provided by the authors to give readers additional information about their work.

© 2023 Odom JN et al. *JAMA Network Open*.

eAppendix. Final Survey

© 2023 Odom JN et al. *JAMA Network Open*.

Building Support for Family Caregivers of Individuals with Cancer

A National Survey of U.S. Cancer Centers

Funded by:

GORDON AND BETTY
MOORE
FOUNDATION

Thank you for participating in this survey about how your cancer center supports family caregivers.

Your effort will help us advocate for and develop national strategies to support the growth of caregiver support programs in U.S. Cancer Centers. Each cancer center's experiences are important and unique. That's why your participation is vital to the success of this effort. **Please help us support family caregivers by filling out this survey and sending it back in the stamped self-addressed envelope or by completing the**

**surv
ey
onli
ne.**

Alabama Alaska Arizona Arkansas California Colorado Connecticut Delaware Florida Georgia Hawaii Idaho Illinois Indiana Iowa
Kansas Kentucky Louisiana
Maine Maryland Massachusetts
Michigan Minnesota Mississippi Missouri Montana Nebraska Nevada
New Hampshire New Jersey New Mexico New York North Carolina North Dakota
Ohio Oklahoma Oregon Pennsylvania Rhode Island South Carolina South Dakota Tennessee
Texas Utah Vermont Virginia Washington West Virginia Wisconsin
Wyoming

© 2023 Odom JN et al. *JAMA Network Open*.

INSTRUCTIONS

- Boxes do not need to be completely filled. Examples of markings that are okay are:

- You are able to complete this survey if:
  1) You have been sent this survey; **AND/OR**
  2) You have a *general knowledge* of support services and programs for family caregivers in your cancer center.

- **We support team efforts!** If you feel uncertain about any responses, we encourage you to ask for help and input from the people you work with.

- If you believe that another individual you work with may be better suited to respond to this survey, no problem! Please call or email Ms. Kayleigh Curry at 205-996-0107 or email her at bettymooresurvey@uab.edu.

- Each cancer center is unique. There are no right or wrong answers. Also, all data from this survey will be reported in aggregate. Individual respondents and cancer centers will NOT be identified in reporting.

- For this survey, a **family caregiver** is defined as a close family member or friend who provides regular support to a patient living with cancer. This person is *not* paid for their support and does not have to live in the same home as the patient.

- Would you rather complete this survey online? No problem!
  Simply go to: https://redcap.link/ccisurvey and follow the instructions.

- **Have questions?** Email or call us!
  Kayleigh Curry, MPH, Program Manager
  Phone: (205) 996-0107
  Email: bettymooresurvey@uab.edu

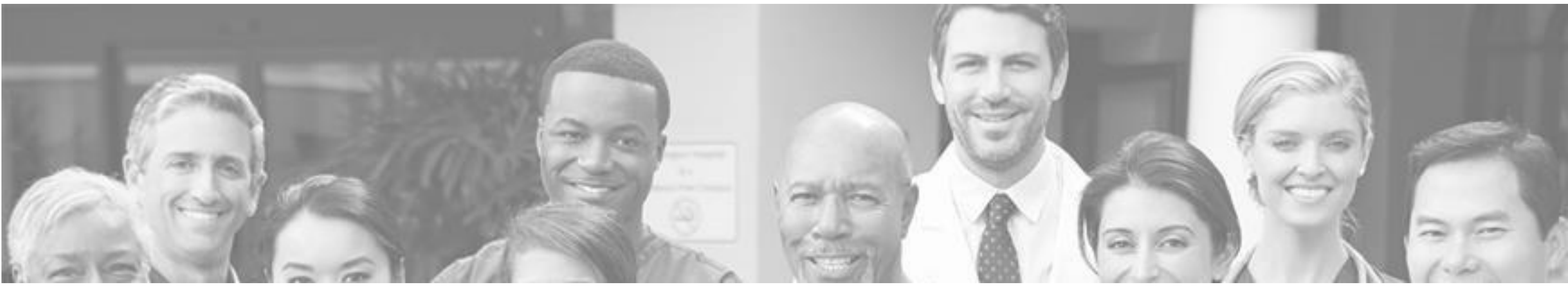

© 2023 Odom JN et al. *JAMA Network Open*.

SECTION A: ABOUT YOU AND YOUR CANCER CENTER

Please fill out the following information:

Cancer Center Name____

Cancer Center ID (found in email under survey link)_________________________

1. How many years have been working at your cancer center in this role?
- ☐ 1 year or less
- ☐ 2-5 years
- ☐ 6-10 years
- ☐ 11-15 years
- ☐ 16-20 years
- ☐ 21 years or more

Please provide approximations for the following questions. We do not expect you to look up this information in detail. If this information is not readily available, please provide your best estimate.

2. What is the approximate number of unique **outpatients** seen in your cancer center **annually**?
- ☐ 1,000 or less outpatients/year
- ☐ 1,000 to 5,000 outpatients/year
- ☐ 5,000 to 10,000 outpatients/year
- ☐ 10,000 to 15,000 outpatients/year
- ☐ 15,000 to 20,000 outpatients/year
- ☐ 20,000 or higher outpatients/year

3. What is your cancer center zip code? ________________________________

4. Approximately how many **total oncologists** (medical, surgical, and radiation) serving adult patients work at your cancer center?
- ☐ 5 or less
- ☐ 6-10
- ☐ 11-15
- ☐ 16-20
- ☐ 21-25
- ☐ 26 or more

© 2023 Odom JN et al. *JAMA Network Open*.

5. Which of the following best describes the ownership of your cancer center?

- ☐ Independently owned (i.e., single hospital or small regional network [up to 3 hospitals] or an independent clinic/physician practice)
- ☐ Hospital, clinic, or physician practice owned by a large regional/multi-state health system that DOES include a health plan
- ☐ Hospital, clinic, or physician practice owned by a large regional/multi-state health system that DOES NOT include a health plan
- ☐ HMO/Payer owned

© 2023 Odom JN et al. *JAMA Network Open*.

☐ Publicly owned (e.g., state, county, city)
☐ Academic medical center (i.e., university-based hospital)
☐ Other (please describe:_______________________________________)

6. Which of the following best describes the population(s) **primarily** served by your cancer center? **(Check all that apply)**

	Rural	Urban	Low Income	Mid-High Income	Don't know/Prefer not to answer
White	☐	☐	☐	☐	☐
Black or African American	☐	☐	☐	☐	☐
Asian	☐	☐	☐	☐	☐
Native Hawaiian or Pacific Islander	☐	☐	☐	☐	☐
American Indian or Alaskan Native	☐	☐	☐	☐	☐
Hispanic or Latino	☐	☐	☐	☐	☐
Other (please specify:__________________)	☐	☐	☐	☐	☐

Next page

SECTION B: YOUR CANCER CENTER'S CAREGIVER PROGRAMS

For the purposes of this survey, we define a **family caregiver program** as a structured, planned, coordinated group of activities and procedures aimed at specifically supporting family caregivers as part of usual care in your cancer center. A program can be aimed at helping caregivers in their role supporting patients and/or in taking care of themselves. A program *can* be one that is offered only for specific cancers (e.g., a support group for caregivers of patients with brain tumors).

<u>Importantly,</u> <u>we do **NOT** consider the following to be a family caregiver program</u>:

© 2023 Odom JN et al. *JAMA Network Open*.

- A program wholly funded by a research study or time-limited quality improvement project.
- Simply having social workers or navigators employed at your cancer center.
- A clinical referral pathway for distressed caregivers that provides services that bills their health insurance (i.e., they become a “patient” with a medical record number in the health system).
- A program focused primarily on patients. A family caregiver program might include patient participation but needs to include the family caregiver as an essential participant in the program.

7. To the best of your knowledge, which of the following types of **family caregiver programs** does your cancer center currently offer?

	No, not offered	Yes, provided onsite at our cancer center	Yes, provided through referral within our greater health system or outside service	Yes, provided both onsite and through referral	Don’t know
a) Individual psychosocial programs for caregivers (e.g., counseling, coping support)	☐	☐	☐	☐	☐
b) Group psychosocial programs for caregivers (e.g., support groups)	☐	☐	☐	☐	☐
c) Peer mentor programs (e.g., matching with another caregiver who has experience with cancer)	☐	☐	☐	☐	☐
d) Complementary and integrative health programs for caregivers (e.g., yoga, massage, acupuncture, music therapy, hypnotherapy)	☐	☐	☐	☐	☐
e) Caregiver-specific distress screening programs (distinct from patient distress screening)	☐	☐	☐	☐	☐
f) Financial or employment-related counseling programs for caregivers	☐	☐	☐	☐	☐
g) Spiritual and/or religious support programs	☐	☐	☐	☐	☐
h) Education classes focused on caregiver self-care/wellness (e.g., exercise, diet/nutrition, sleep, stress management)	☐	☐	☐	☐	☐
i) Caregiver specific training or education classes focused on medical and/or nursing tasks (e.g., medication and symptom management, wound care)	☐	☐	☐	☐	☐

8. ***(continued)***

	No, not offered	Yes, provided onsite at our cancer center	Yes, provided through referral within our greater health system or outside service	Yes, provided both onsite and through referral	Don’t know
j) Information and referral services (programs that help caregivers identify local resources, including telephone helplines)	☐	☐	☐	☐	☐

© 2023 Odom JN et al. *JAMA Network Open*.

k) Programs for caregivers age 18 or less providing care to a family member with cancer	☐	☐	☐	☐	☐
l) Other programs for caregivers (Please describe)______________________________ ______________________________ ______________________________	☐	☐	☐	☐	☐

If you answered "No, not offered" and/or "Don't know" to ALL items (a through k) in Question 6, then SKIP Questions 9-13.

8. Family caregiver programs may be selected and adapted from many different sources. Thinking about how your family caregiver programs were started at your cancer center, please select the top 3 sources that influenced their development: **(Check up to 3 boxes)**

- ☐ A journal
- ☐ A conference
- ☐ A colleague
- ☐ Another cancer center
- ☐ A cancer organization (Please specify:_________________________)
- ☐ Someone in your community
- ☐ An internet website (Please describe:__________________________)
- ☐ Other (Please describe:___________________________)
- ☐ Don't know

9. Family caregiver programs may be funded in many different ways. Thinking about how family caregiver programs are paid for at your cancer center, please select the top 3 ways by which they are funded: **(Check up to 3 boxes)**

- ☐ Patient's health insurance
- ☐ Caregiver's health insurance
- ☐ Philanthropic or other individual or community donations
- ☐ Cancer center or hospital supported
- ☐ Caregivers pay out of pocket
- ☐ State and/or federal funding
- ☐ Grant
- ☐ Other funding mechanism (Please describe:_____________________________)

10. Thinking in general about the family caregiver programs offered by your cancer center, why were they chosen? **(Check all that apply)**

- ☐ We had used it (or something like it) before
- ☐ People in the community requested this type of program
- ☐ There was scientific evidence saying that the program works
- ☐ It was easy to implement

© 2023 Odom JN et al. *JAMA Network Open*.

☐ It was available for free or low cost
☐ Cancer center leaders encouraged us to use this program
☐ The program fit our budget
☐ Other cancer centers like ours are using this program
☐ We felt it was better than the alternatives
☐ We did not know of any alternatives
☐ We had a champion or leader behind the program
☐ We had staff in strong support of the program
☐ Technical assistance was available to help us with the program
☐ Other (Please describe:____)
☐ Don't know why they were chosen

11. Has your cancer center ever received any awards or recognition for your family caregiver programs?
☐ Yes (Please describe:____)
☐ No
☐ Don't know

12. In your opinion, to what extent are your cancer center's clinicians aware of its family caregiver programs, from 1 (Not at all aware) to 10 (Extremely aware):

☐	☐	☐	☐	☐	☐	☐	☐	☐	☐
1	2	3	4	5	6	7	8	9	10

Not at all aware ←→ Extremely aware

Next page ➡

SECTION C: SUPPORT FOR DEVELOPING CAREGIVER PROGRAMS

13. If a **new family caregiver program** were going to be established in your cancer center, how important would each of the following characteristics of the program be?

	Not at all important	Slightly important	Moderately important	Very important	Extremely important

© 2023 Odom JN et al. *JAMA Network Open*.

a) Easy to implement	☐	☐	☐	☐	☐
b) Available for free	☐	☐	☐	☐	☐
c) Has been shown to work	☐	☐	☐	☐	☐
d) Other cancer centers are using it	☐	☐	☐	☐	☐
e) Easy to evaluate	☐	☐	☐	☐	☐
f) Used in populations like ours	☐	☐	☐	☐	☐
g) Low cost and/or cost effective	☐	☐	☐	☐	☐
h) Reduces costs	☐	☐	☐	☐	☐
i) Reduces patient healthcare utilization (e.g., lower readmission rates)	☐	☐	☐	☐	☐
j) Consistent with our cancer center's mission and strategic plan	☐	☐	☐	☐	☐
k) Consistent with our cancer center's image	☐	☐	☐	☐	☐
l) Technical assistance available	☐	☐	☐	☐	☐
m) Innovative	☐	☐	☐	☐	☐
n) Able to be used on a trial basis	☐	☐	☐	☐	☐
o) Results could be easily observed	☐	☐	☐	☐	☐
p) Providers and staff actively support and promote the program	☐	☐	☐	☐	☐
q) Clinic leadership actively supports and promotes the program	☐	☐	☐	☐	☐
r) Helps our cancer center meet quality indicators	☐	☐	☐	☐	☐
s) Helps our cancer meet accreditation requirements	☐	☐	☐	☐	☐
t) Other (Please describe:____________ ______________________________ ______________________________)	☐	☐	☐	☐	☐

14. If you found a family caregiver program that had been published in a journal, how confident do you feel that you and your colleagues could adapt it to fit your community's needs? (e.g., tailoring it to the culture of the local community)

- ☐ Not at all confident
- ☐ Not very confident
- ☐ Neutral
- ☐ Somewhat confident
- ☐ Totally confident

15. Which of the following types of training would help your cancer center select, adapt, and implement family caregiver programs: **(Check all that apply)**

- ☐ How to involve other stakeholders/partners
- ☐ How to assess and utilize available personnel and resources
- ☐ How to find and secure funding

© 2023 Odom JN et al. *JAMA Network Open*.

- ☐ How do identify evidence-based family caregiver program models
- ☐ How to obtain program materials (e.g., educational/promotional materials, participant incentives, etc.)
- ☐ How to train clinicians and staff to implement the program
- ☐ How to adapt a program and materials for cultural appropriateness (e.g., context, region, language, ethnicity, culture, religion)
- ☐ How to identify what program aspects can and cannot be changed
- ☐ How to pilot test a program with the intended audience
- ☐ How to develop an implementation and evaluation plan
- ☐ How to enroll family caregivers into the program
- ☐ How to evaluate program outcomes
- ☐ How to document and track the family caregiver program
- ☐ No training is needed
- ☐ Other (please describe): ________________________________

16. If comprehensive training, technical assistance, and coaching could be provided to support the selection, implementation, and maintenance of family caregiver programs, how much do you think your cancer center would be willing to pay for these services?

- ☐ >$20,000/year
- ☐ $15,000-$20,000/year
- ☐ $10,000-$15,000/year
- ☐ $7,500-$10,000/year
- ☐ $5,000-$7,500/year
- ☐ $2,500-$5,000/year
- ☐ $1,000-$2,500/year
- ☐ <$1,000/year
- ☐ I don’t think our cancer center would be willing to pay

Next page ➡

17. Of the following areas that family caregivers may find challenging, which **5** should family caregiver programs be focused on helping the most? **(Check up to 5 boxes)**

© 2023 Odom JN et al. *JAMA Network Open*.

- ☐ Transportation
- ☐ Housing/living arrangements
- ☐ Finances/insurance
- ☐ Understanding patient's health condition, including prognosis and what to expect

© 2023 Odom JN et al. *JAMA Network Open*.

☐ Work/Employment
☐ Effort assisting with activities of daily living
☐ Coordinating services
☐ Engaging in social activities
☐ Caregiver's relationship with the patient
☐ Caregiver's relationship with other family, friends, children
☐ Intimacy
☐ Spiritual distress
☐ Managing emotions and stress
☐ Taking care of one's own health while caring for the patient
☐ Understanding medical procedures, tests, treatments
☐ Managing patient's symptoms
☐ Managing and administering medications
☐ Nutrition/meal preparation
☐ Communicating with health professionals
☐ Planning for the future and/or advance care planning
☐ Helping patients make healthcare and other life decisions
☐ Knowing how and when to ask for and seek help
☐ Other (Please describe:________________ ____)
☐ Other (Please describe:________________ ____)

19. Which and how many of the following individuals helped you in any way complete this survey? **(Check all that apply and include a number for how many)**

☐ Behavioral health counselor (How many?____)
☐ Health educator (How many?____)
☐ Physician (How many?____)
☐ Nurse practitioner, physician assistant, DO (How many?____)
☐ Nurse (including nurse navigators and nurse educators) (How many?____)
☐ Social worker (How many?____)
☐ Psychologist (How many?____)
☐ Psychiatrist (How many?____)
☐ Healthcare administrator, coordinator, or director (How many?____)
☐ Human resources professional (How many?____)
☐ Other (Please describe:____________________________) (How many?____)

20. What is the date you completed this survey? **_______/______/20_______**

21. Would you be willing to participate in a 20-30 minute telephone interview about your responses and about how we can develop training programs to help cancer centers identify and implement family caregiver programs?

☐ Yes
☐ No

© 2023 Odom JN et al. *JAMA Network Open*.

If yes, please tell us:
Your name:____
Title:____
Email:____
Best contact telephone number:_____________________________

22. We would like to send you a $20 Visa gift card as a token of appreciation for completing this survey. Please provide the following information to receive your card:

Last name:______________________________First name_______________________________

DOB (**required for gift card**): __________________________

Mailing address (where Visa gift card will be sent):

Street:________________________________City/Town:________________Zip code:__________

Email (optional):___________________________Cell phone (optional):______________________

23. What else would you like to tell us about family caregiver programs at your cancer center or other comments in general?
____
____
____
____
____
____
____
____
____
____
____
____
____
____
____
____

*******END OF SURVEY*******

© 2023 Odom JN et al. *JAMA Network Open*.

THANK YOU VERY MUCH for being part of this important national survey. We greatly appreciate your time. Your responses are important, and we are glad to have your cancer center represented in our effort to support family caregivers.

OPTION FOR RETURNING THE SURVEY

(1) Scan the survey: Email back to us at kayleigh@uab.edu

(2) Mail the survey: Send to the following address:

1720 2nd Ave S, MT 418 | Birmingham, AL 35294-4410

*Need a stamp and envelope? Call us at (205) 542-6879 or email Kayleigh Curry at kayleigh@uab.edu

ALSO, please remember to put your cancer center ID on the survey (included in our email to you). Thank you!

© 2023 Odom JN et al. *JAMA Network Open*.

eFigure 1. Study Flow Diagram

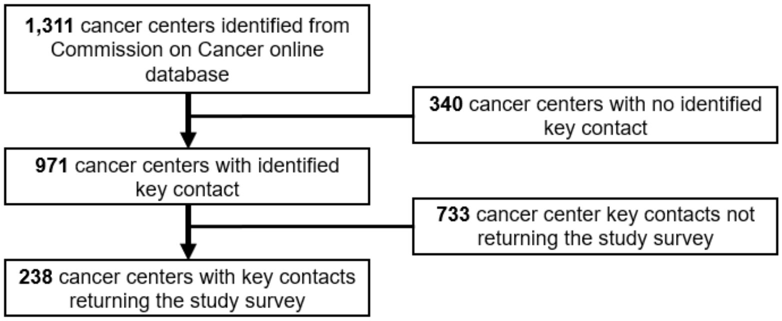

© 2023 Odom JN et al. *JAMA Network Open*.

eFigure 2. Weight-Adjusted Primary Sources of Funding for Family Caregiver Programs (N = 238)

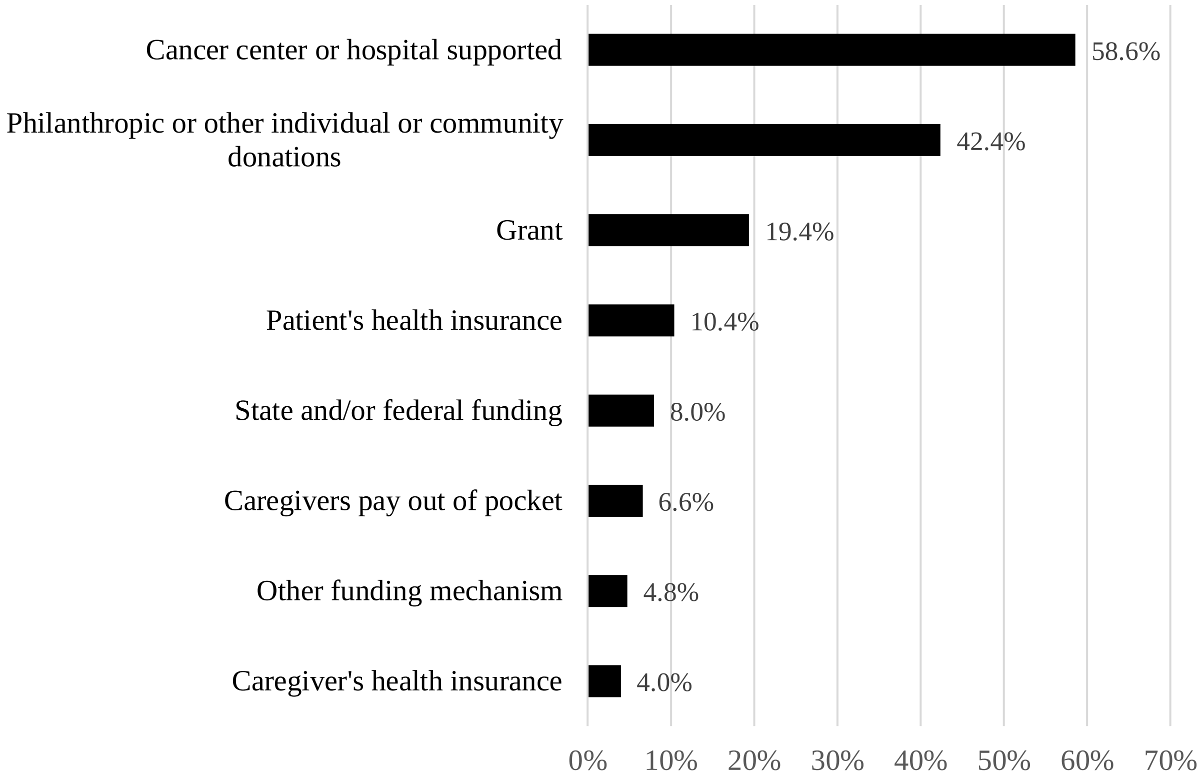

*Survey item was a "check up to 3 options"

© 2023 Odom JN et al. *JAMA Network Open*.

eTable. Nonresponding vs Responding Cancer Center Characteristics

**Characteristic**	**Non-responding cancer centers (N=733)**		**Responding cancer centers (N=238)**		***p*-value***	**Cramer's V**
	No.	**%**	No.	%		
U.S Region						
West	137	18.7	47	19.8	.220	.067
Midwest	198	27.0	70	29.4		
Northeast	127	17.3	50	21.0		
South	271	37.0	71	29.8		
Geography						
Rural	645	88.0	195	81.9	.017	.076
Urban	88	12.0	43	18.1		
*Pearson Chi-Square						

© 2023 Odom JN et al. *JAMA Network Open*.
