# Supplementary material for: Availability of Family Caregiver Programs in US Cancer Centers
Source: JAMA Netw Open. 2023 Oct 11;6(10):e2337250. doi: 10.1001/jamanetworkopen.2023.37250 (PMC10568368; doi:10.1001/jamanetworkopen.2023.37250)
Supplement: Supplement 2. — Data Sharing Statement [file jamanetwopen-e2337250-s002.pdf]

Data Sharing Statement

Odom. Availability of Family Caregiver Programs in US Cancer Centers. *JAMA Netw Open*. Published October 11, 2023. doi:10.1001/jamanetworkopen.2023.37250

Data
Data available: No

Additional Information
Explanation for why data not available: De-identified data will be made available upon reasonable request from the corresponding author.
